# Case Report: Allogeneic adipose-derived mesenchymal stem cells for severe chronic kidney disease in a dog

**DOI:** 10.3389/fvets.2026.1977920

**Published:** 2026-10-07

**Authors:** Yu Yu, Xuemin Lv, Xinyuan Zhou, Yuxin Ma, Hao Li, Weiwei Wang, Yizhe Song

**Affiliations:** 1 College of Life Sciences, Yantai University, Yantai, Shandong, China; 2 OUR PET Hospital, Shanghai, China

**Keywords:** adipose-derived mesenchymal stem cells, allogeneic stem cell therapy, canine chronic kidney disease, renal fibrosis, renal regenerative medicine

## Abstract

This case report explored the clinical improvement and short-term tolerability of allogeneic adipose-derived mesenchymal stem cells (AD-MSCs) in the treatment of severe chronic kidney disease (CKD) in dogs. A 9-year-old female Pomeranian was diagnosed with chronic kidney disease (CKD) caused by acute kidney injury and, after conventional treatment failed, received intravenous infusion of AD-MSCs from healthy donor dogs. The AD-MSCs were aseptically collected, isolated, cultured, characterized, and subjected to microbiological safety testing, and were then administered intravenously at a dose of 3 × 10^6^cells/kg every 2 weeks for a total of four infusions, with dexamethasone premedication to reduce the risk of adverse reactions. Following AD-MSC administration and continued conventional care, reductions in serum creatinine and phosphorus and a downward trend in blood urea nitrogen were observed, along with improvements in body weight, body condition score, appetite, and mental status; no adverse events were observed. The results of this case suggest the feasibility and short-term tolerability of allogeneic AD-MSCs for severe CKD in dogs and provide a reference for clinical research on cell-based therapy for canine CKD.

## 1 Introduction

Chronic kidney disease (CKD) and its acute-on-chronic kidney disease (ACKD) are highly prevalent in canine clinical practice. CKD is defined as structural or functional abnormalities of one or both kidneys that persist for an extended period, typically 3 months or longer (1). Despite its diverse etiologies, including familial, congenital and acquired factors, canine CKD ultimately leads to a reduction in total glomerular filtration rate (GFR) and disruption of systemic homeostasis (2). The prevalence of canine CKD varies widely in global reports, ranging from 0.05 to 3.74% (3). Identified risk factors include advanced age, specific breeds, small body size, periodontal disease, and concurrent heart disease as a critical comorbidity (4). Prominent clinical signs of canine CKD include halitosis, weight loss, polyuria, polydipsia, urinary incontinence, vomiting, anorexia, lethargy and diarrhea (5, 6). Renomegaly may occasionally occur in CKD cases complicated by renal neoplasia, pyelonephritis or ureteral obstruction (7). Biochemical abnormalities include azotemia with inadequately concentrated urine, metabolic acidosis and hyperphosphatemia. Additionally, some patients may present with hypokalemia, non-regenerative anemia, hypoalbuminemia, dyslipidemia and bacterial urinary tract infection (8, 9). The International Renal Interest Society (IRIS) has proposed a four-stage staging system for canine CKD based on serum biochemistry, urinalysis results and systemic arterial blood pressure. The IRIS stages range from mild (Stage 1) to most severe (Stage 4), which standardizes the classification of CKD cases to facilitate diagnosis, treatment, prognosis and research (10). The treatment of CKD aims to correct metabolic imbalances and slow disease progression, and requires lifelong management as CKD is an irreversible condition (11). Conservative management of CKD consists of supportive and symptomatic therapy, including conventional medical treatment and renal replacement therapy (12). The core goals of canine CKD management include alleviating uremic clinical signs and improving quality of life, reducing metabolic and electrolyte disturbances (including water, phosphorus, potassium and acid–base imbalances), providing adequate nutrition through dietary modification with sufficient calories and essential amino acids (13), while limiting phosphorus and protein intake to slow disease progression, and retarding the progression of renal failure by controlling hypertension, proteinuria and secondary metabolic complications (14). Renal replacement therapy, such as hemodialysis for end-stage renal disease (ESRD), removes urea, creatinine and excess fluid through diffusion and ultrafiltration via a dialysis machine and membrane, but this approach is extremely limited in clinical canine practice due to technical and economic constraints (15). Therefore, emerging evidence has demonstrated that mesenchymal stem cells (MSCs) are closely associated with renal repair. MSCs are multipotent stem cells derived from the mesoderm, which are widely distributed in tissues such as bone marrow, adipose tissue, umbilical cord and placenta (16). MSCs possess multidirectional differentiation potential, immunomodulatory effects, tropism for injured tissues, paracrine activity and low immunogenicity. Renal interstitial fibrosis is one of the key mechanisms driving CKD progression, and MSCs can block the fibrotic process through multiple pathways: they inhibit the transformation of renal tubular epithelial cells and glomerular mesangial cells into myofibroblasts by blocking the TGF-β/Smad and other fibrotic pathways (17), reduce the activation and proliferation of myofibroblasts to decrease extracellular matrix (ECM) production from the source, and suppress the expression of key profibrotic factors such as TGF-β1 and connective tissue growth factor (CTGF) (18), thereby blocking the fibrotic cascade and delaying the progression from nephritis to chronic kidney disease (19).

However, this is a single-case observational report. The clinical and biochemical changes observed during follow-up occurred in the context of continued conventional therapy, dexamethasone premedication, comorbidities, and the absence of a control group. No renal histopathological or mechanistic analyses were performed, and the follow-up period was short. Therefore, this report should be regarded as providing preliminary observations on feasibility and short-term tolerability, rather than as evidence of therapeutic efficacy, safety, renal regeneration, or disease modification. Controlled studies with protocolized follow-up are needed to determine whether AD-MSCs provide clinical benefit in canine CKD.

## 2 Case description

In September 2024, a 9-year-old female spayed Pomeranian dog presented with anorexia, nausea, vomiting and lethargy. On physical examination, the dog weighed 2.3 kg, had a body condition score (BCS) of 4/9, and pale mucous membranes. Laboratory findings revealed serum creatinine (SCr) of 106 μmol/L (IRIS Stage 1 reference interval: <125 μmol/L), blood urea nitrogen (BUN) of 18.6 mmol/L (reference interval: 5.7–12.9 mmol/L), symmetric dimethylarginine (SDMA) of 18 μg/dL (reference interval: <18 μg/dL). Abdominal ultrasound showed bilateral renal degenerative changes with multiple calcifications, urolithiasis, and approximately 2 mm cortical cystic nodules. Renal function testing indicated early renal injury with abnormal glomerular filtration function, complicated by degenerative valvular heart disease and chronic cholecystitis with biliary sludge accumulation. Over the following 3 months, the dog’s serum creatinine remained within the normal range, SDMA remained stable at 18 μg/dL, and BUN had also shown a downward trend. However, by the fourth month, the renal indices deteriorated rapidly, with all parameters rising markedly, and signs of acute kidney injury developed. From February 2025 to April 2025, the patient repeatedly presented to the emergency department for acute vomiting and progressive renal dysfunction, with sustained elevation of creatinine (>125 μmol/L) and SDMA (>18 μg/dL) above the upper reference limit, indicating progression from early renal injury to overt renal insufficiency. In May 2025, the patient developed vomiting and loose stools and was diagnosed with gastroenteritis accompanied by further deterioration of renal function. SCr increased to 160 μmol/L (IRIS stage 2 reference range: 125–250 μmol/L), and SDMA increased to 30 μg/dL (IRIS stage 2 reference range: 18–35 μg/dL). Ultrasound revealed progression of bilateral renal cystic nodules, and blood gas analysis showed metabolic acidosis and hyperkalemia, indicating that CKD had progressed to stage 2 (Table 1).

**Table 1:** Renal parameters and clinical course.

TIME	SCr (μmol/L)	BUN (mmol/L)	SDMA (μg/dL)	Clinical signs	Treatment	CKD stage
0	106	18.6	18	Occasional vomiting; cataract; BW 2.5 KG	Regular check-ups; owner-reported self-administered medication	/
40	97	19.1	12	Soft stool; picky eating; BW 2.2 KG	Continue medication; regular follow-up	/
115	83	11.6	16	Poor spirit; decreased appetite; reduced activity	Continue medication; regular follow-up	/
129	152	>46.4	18	Vomiting; diarrhea; BW 2.3 KG	AKI hospitalization; LRS fluids; exercise restriction; renal diet; avoid nephrotoxic drugs; monitor urine output, biochemistry, blood gas	AKI
159	159	16.3	30	Vomiting; trembling during sleep; BW 2.2 KG	Antiemetic; gastroprotection; dietary management	CKD stageII
230	160	16.8	/	Several episodes of vomiting; soft stool; BW 2.2 KG	Symptomatic treatment for gastroenteritis; antiemetics; gastroprotection; diet; fluids	CKD stageII
264	160	17.8	/	Vomited undigested food; vomited yellow water; soft stoo; BW 2.3 KG	CKD management; AD-MSC therapy initiated	CKD stageII
338	136	14.4	/	Decreased appetite; decreased mental status; BW 2.4 KG	CKD management; AD-MSC treatment completed	CKD stageII
419	143	14.2	18	General follow-up; grade 3 heart murmur; BW 2.5 KG	CKD management	CKD stageII
460	137	13.7	/	Follow-up for renal indicators; BW 2.7 KG	CKD management	CKD stageII
481	124	10.8	16	Follow-up; trembling at home; BW 2.6 KG	CKD management	CKD stage I
508	129	14.0	16	Decreased appetite; soft stool; BW 2.6 KG	CKD management	CKD stage I

During this period, the patient was switched to a veterinary renal prescription diet with strict restriction of protein, phosphorus and sodium intake, supplemented with omega-3 fatty acids and antioxidants to slow CKD progression. Fresh drinking water was provided ad libitum 24 h a day. All nephrotoxic drugs were strictly avoided, and Angiotensin-Converting Enzyme Inhibitor/Angiotensin Receptor Blocker drugs were used with caution, with continuous monitoring of azotemia during medication. However, because this was a retrospective case report, complete drug records (including exact drug names, doses, routes, frequencies, and durations) were not available for all treatments. This limitation is acknowledged. Despite the supportive care provided, the patient’s renal function continued to deteriorate. The patient developed acute exacerbation of CKD with severe renal impairment, acid–base and electrolyte disorders, and showed no improvement in physical examination or biochemical parameters. Therefore, with the informed consent of the pet owner, MSC therapy was initiated in June 2025 to prevent further renal damage from CKD, alleviate clinical signs, and improve the patient’s quality of life.

Adipose tissue was aseptically collected from a healthy donor dog with normal hematological parameters and negative infectious disease screening results. The collected samples were washed 3 times with phosphate-buffered saline (PBS, Servicebio, China), then placed in a PBS centrifuge tube containing 1% penicillin–streptomycin (P/S, Gibco, USA) and transferred to a laminar flow hood for subsequent processing (Figure 1A).

**Figure 1:**
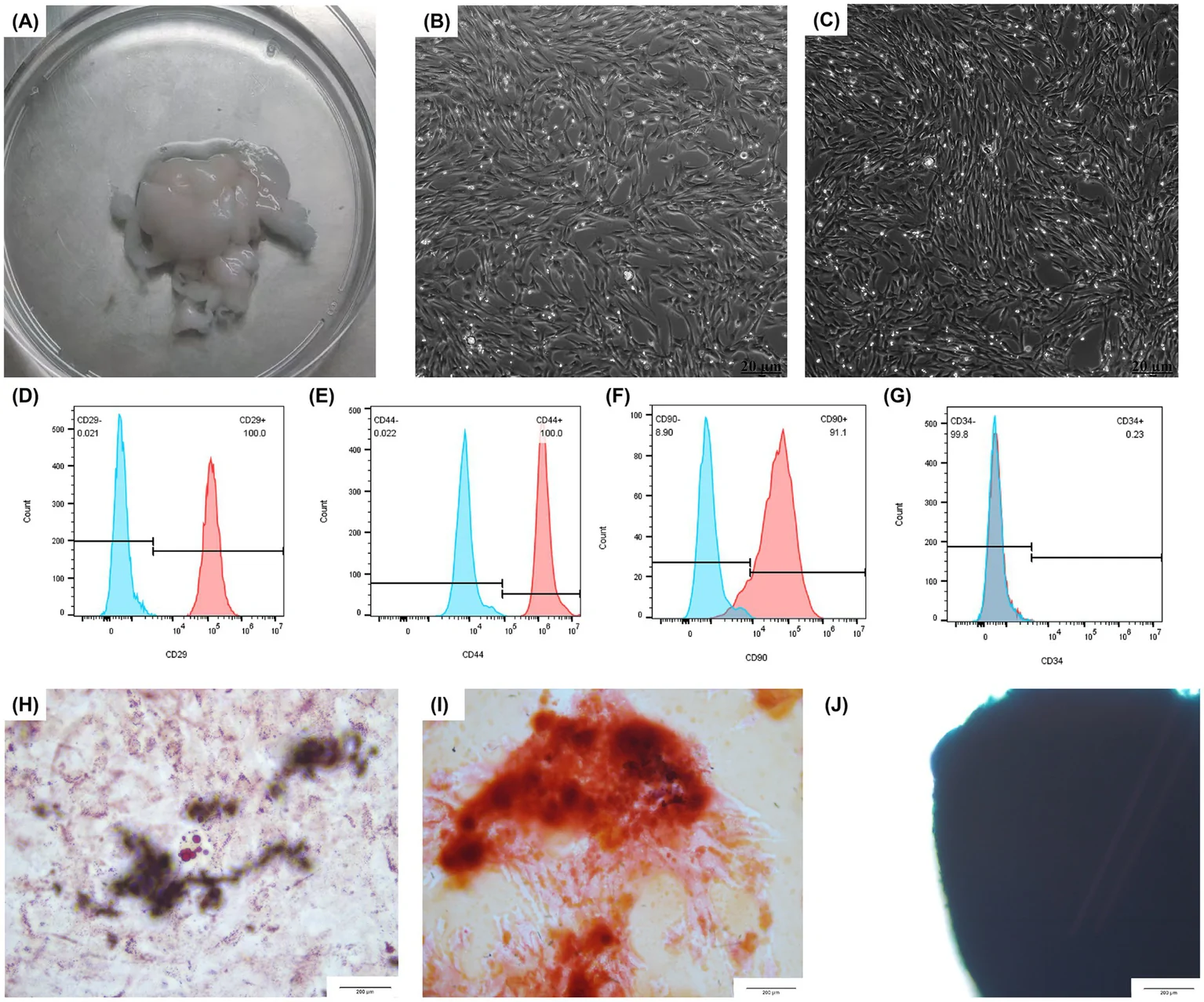
Canine adipose tissue and characterization of AD-MSCs. **(A)** Canine adipose tissue. **(B,C)** At × 100 magnification, canine AD-MSCs exhibited a spindle-shaped morphology. Scale bars: 20 μm. **(D–G)** Representative flow cytometric histograms of CD29, CD44, CD90, and CD34 expression on AD-MSCs. Blue histograms represent matched isotype controls, and red histograms represent specific antibody staining. The percentages of positive cells are indicated. A total of 10,000 events were acquired per sample, and viable cells were gated based on 7-AAD negativity. **(H)** Adipogenic differentiation evaluated by Oil Red O staining. **(I)** Osteogenic differentiation evaluated by Alizarin Red staining. **(J)** Chondrogenic differentiation evaluated by Alcian Blue staining. These immunophenotypic characteristics, together with the typical spindle-shaped morphology and trilineage differentiation potential, confirmed that the isolated cells met the criteria for mesenchymal stem cells.

The adipose tissue was sequentially immersed in 3 centrifuge tubes containing 75% ethanol for 3 s per tube, then transferred to a Petri dish containing PBS to remove blood clots. The treated adipose tissue was then transferred to a new centrifuge tube, an appropriate amount of PBS was added, the tube was gently inverted to mix, and the PBS was discarded; this washing step was repeated for 10 cycles. The washed adipose tissue was transferred to a new centrifuge tube with sterile forceps and cut into 0.5–1 mm^3^ tissue fragments with surgical scissors.

Type I collagenase (1 mg/mL, Sigma-Aldrich, USA) was added to the centrifuge tube, and the mixture was incubated in a 37 °C-water bath with continuous stirring for 45 min to ensure sufficient digestion of adipose tissue. After digestion, an equal volume of complete medium was added to neutralize collagenase activity. The complete medium was prepared with Dulbecco’s Modified Eagle Medium (DMEM, BasalMedia Technologies, China), supplemented with 10% fetal bovine serum (FBS, Excell, China), 1% penicillin–streptomycin (P/S), and 5 ng/mL basic fibroblast growth factor (bFGF, Xinhe Biotechnology Co., Ltd., China).

The cell suspension was repeatedly pipetted until fibrous aggregates were completely dispersed, then filtered through a 100 μm cell strainer (pluriSelect, Germany) to remove undigested tissue debris and cell clumps. After centrifugation, the cell pellet was resuspended in the prepared DMEM complete medium, plated in 6-well plates, and aseptically cultured for 7 days at 37 °C in a 5% CO₂ incubator, with medium changed twice during the culture period. Cells were continuously cultured until adherent cell confluence reached 70–80%, then subcultured. Cells were cryopreserved after 5 consecutive passages.

Morphological observation under a microscope showed that the isolated and cultured MSCs exhibited typical spindle-shaped, fibroblast-like morphological characteristics (Figures 1B,C). Flow cytometry was performed to identify the immunophenotype of the canine AD-MSCs. The directly conjugated antibodies used in the experiment were as follows: PE-conjugated CD29 antibody (clone TS2/16, cat. no. 303004, BioLegend, USA), FITC-conjugated CD44 antibody (clone IM7, cat. no. MA1-10229, Invitrogen, USA), PE-conjugated CD90 antibody (clone 5E10, cat. no. 555596, BD Biosciences, USA), and PE-conjugated CD34 antibody (clone 1H6, cat. no. 559369, BD Biosciences, USA). Isotype controls were set as follows: PE-conjugated mouse IgG1, κ isotype control (clone MOPC-21, cat. no. 555749, BD Biosciences, USA), FITC-conjugated rat IgG2b κ isotype control (clone eB149/10H5, cat. no. 11–4,031-82, Invitrogen, USA), and unconjugated mouse IgG1 κ isotype control (clone P3.6.2.8.1, cat. no. 14–4,714-82, Invitrogen, USA).

Cells were detected using a CytoFLEX flow cytometer (Beckman Coulter, USA), and the data were analyzed using FlowJo software (Tree Star, USA; Figures 1D–G). Flow cytometric characterization was performed using passage 5 AD-MSCs. Cells were harvested, washed with PBS, and incubated with the indicated antibodies or matched isotype controls. A total of 10,000 events per sample were acquired on a CytoFLEX flow cytometer (Beckman Coulter, USA) and analyzed using FlowJo software (Tree Star, USA). The gating strategy was as follows: (1) FSC-A/SSC-A gate to exclude debris; (2) FSC-A/FSC-H gate to exclude doublets; (3) 7-AAD-negative gate to select viable cells; and (4) marker expression was evaluated within the viable cell gate using isotype controls to set positive thresholds. Cell viability before infusion was >90%. Flow cytometric analysis showed that AD-MSCs were highly positive for CD29 (96.7%), CD44 (99.6%), and CD90 (91.5%), and negative for CD34 (0.23%; Figures 1D–G). A total of 10,000 events were acquired for each sample, and the viability of the analyzed cells was >90%. Representative isotype controls are shown as blue histograms. In addition, MSCs were inoculated into corresponding differentiation media, and the results confirmed their trilineage differentiation potential into adipocytes, osteoblasts and chondrocytes, further verifying that the isolated cells were mesenchymal stem cells (Figures 1H–J).

Cryopreserved AD-MSCs at passage 4 (P4) were thawed and cultured until reaching 80–90% confluency; the resulting passage 5 (P5) cells were used for infusion. Cells were then digested with TrypLE™ Express enzyme (1X, Red; Gibco, USA). After digestion, cells were washed with PBS to completely remove residues from the culture process. Cell viability was assessed by trypan blue exclusion after thawing and immediately before infusion; the viability was 97.6%. Microbiological quality control included tests for bacterial and fungal contamination, mycoplasma, and endotoxin, all of which were negative. The treated cells were resuspended in 1 mL of cell cryopreservation premix (Selcell, China), which is free of dimethyl sulfoxide (DMSO) and animal-derived components. The cell cryopreservation premix was pre-validated by our laboratory, demonstrating the capability to maintain cell viability above 90% for up to 72 h at 2–8 °C. The cell suspension was immediately transferred to a sterile vial and stored at 2–8 °C in the dark, with infusion completed within 24 h. Before infusion, the cell suspension was aspirated and added to an infusion bottle containing 20 mL of normal saline, with a final infusion dose of 6.9 × 10^6^ cells/20 mL (cell viability: 95.8%, equivalent to 3 × 10^6^ cells/kg,). The infusion was administered slowly via an intravenous (IV) catheter over 60 min. To reduce the risk of infusion reactions, the patient was pre-medicated with dexamethasone (0.5 mg/kg IV) prior to infusion. The patient’s vital signs were continuously monitored throughout the infusion and for 30 min after the end of the infusion, with observation extended to 24 h post-infusion. Infusions were administered every 2 weeks for a total of 4 doses, with the last infusion administered on July 29, 2025. At the two-month follow-up after the final cell infusion, serum creatinine had decreased to 136 μmol/L, and the BUN concentration showed a sustained downward trend, reaching 14.4 mmol/L (Figure 2). Over the course of treatment, the dog showed some improvement in mental status and appetite. However, because conventional therapy was continued and dexamethasone was administered before each infusion, these improvements cannot be attributed solely to AD-MSCs.

**Figure 2:**
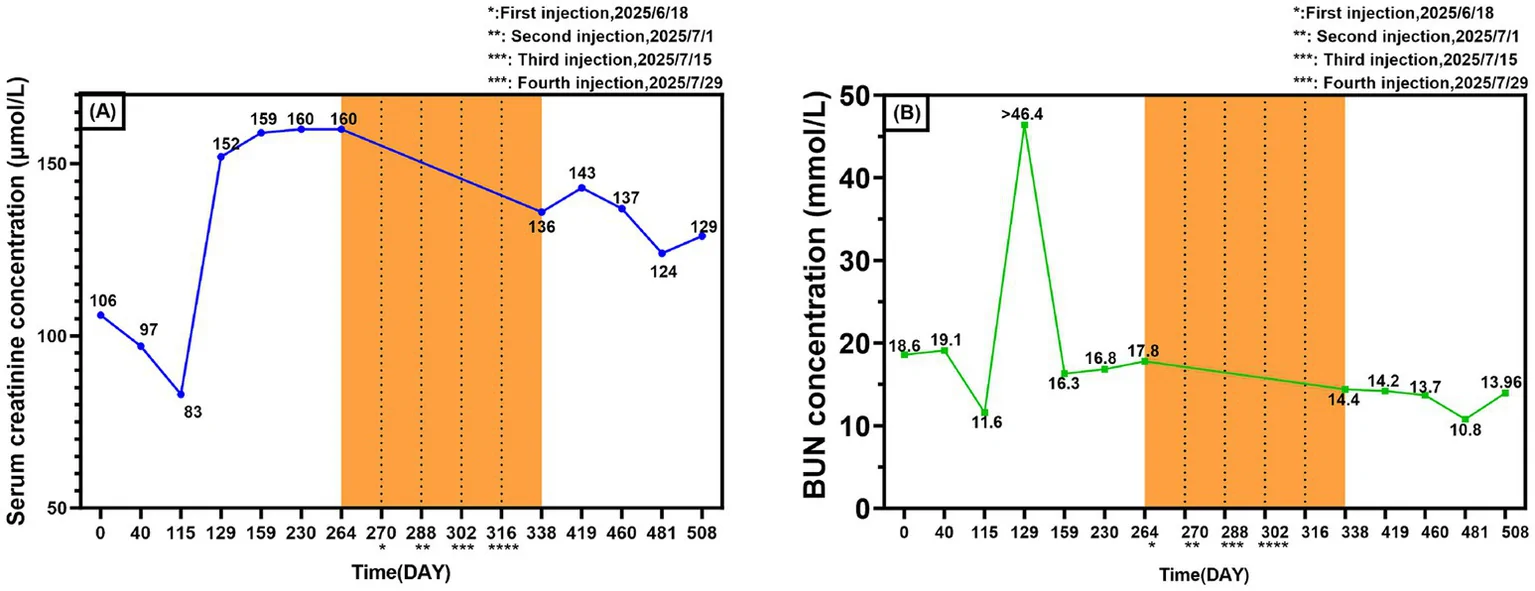
Serial changes in serum creatinine (SCr, μmol/L) and blood urea nitrogen (BUN, mmol/L) throughout the disease course. The yellow-highlighted region indicates the time points after initiation of mesenchymal stem cell (MSC) therapy (June 2025) and the subsequent follow-up period. Following MSC treatment, SCr progressively declined to within the normal reference interval, and BUN showed a sustained downward trend, reflecting an improvement in renal function.

During the subsequent follow-up, multidimensional assessments were performed on the dog, including physical examination (PE), complete blood count (CBC) and serum biochemical testing. The assessment results showed clinically apparent improvements across multiple key indicators: on physical examination, the dog’s body weight and body condition score (BCS) both increased, and the mucous membrane color returned to normal; serum biochemical testing revealed a downward trend in both creatinine and phosphorus levels; inflammation-related indicators including total white blood cell count, neutrophils and lymphocytes were all within the normal range, with no pathological inflammatory hemogram; red blood cell count, hemoglobin and related indicators were normal; all platelet parameters were within normal limits, and no coagulation or thrombosis-related abnormalities were observed, suggesting a stable systemic hematological status at this assessment (Table 2).

**Table 2:** Longitudinal CBC data before and after MSC therapy.

TIME	WBC (×10^9^/L)	RBC (×10^12^/L)	HGB (g/L)	HCT (%)	MCV (fL)	MCH (pg)	MCHC (g/L)	PLT (×10^9^/L)	LYM (%)	EO (%)	LYM (×10^9^/L)	NEU#(×10^9^/L)
0	10.17	6.94	168.0	/	72.51	24.1	334	420	0.26	0.03	2.6	6.70
115	12.86	6.34	152.0	44.13	69.53	23.9	345	321	0.15	0.01	1.9	10.43
129	11.73	5.44	125.9	38.79	71.35	23.16	324.6	315	11.09	1.41	1.3	11.55
159	9.82	5.83	138.7	42.43	72.78	23.79	326.9	384	15.53	0.97	1.5	7.97
230	10.99	6.4	160.0	46.50	72.7	24.9	344	514	0.208	0.02	2.3	8.04
264	9.64	6.49	165.7	43.84	67.56	25.53	377.9	156	16.75	1.21	1.6	7.61
338	11.88	6.91	166.9	48.05	69.54	24.16	347.4	353	21.23	2.76	/	8.45
419	13.43	6.93	171.8	49.39	71.28	24.79	347.7	357	20.65	2.69	2.8	9.74
460	19.30	8.13	201.3	56.46	69.41	24.75	356.5	293	11.74	1.71	2.3	11.95
481	13.07	6.09	152.3	43.03	70.72	25.03	353.9	283	9.50	1.79	1.2	10.96
508	13.71	6.45	161.9	47.66	73.93	25.10	339.6	171	8.46	2.07	1.2	/

No significant adverse reactions were observed throughout the entire treatment process, indicating that the dog had a consistent positive response to this treatment regimen and that it had a favorable safety profile. In summary, this case fully highlights the application potential of this treatment regimen and also provides a valuable reference for clinical research and treatment of similar cases of chronic kidney disease in the future.

## 3 Discussion

This case report describes the clinical efficacy of AD-MSCs in the treatment of severe canine chronic kidney disease (CKD), and provides a clinical reference for stem cell-based therapy for canine CKD. Canine CKD is a common irreversible chronic disease in clinical practice. Although conventional conservative treatment can temporarily relieve clinical symptoms, it is difficult to reverse the progression of renal injury. Especially for drug-induced acute-on-chronic CKD, where traditional treatment modalities often fail to achieve satisfactory therapeutic effects (14). The core pathological changes of canine CKD are renal interstitial fibrosis and progressive nephron injury (20), a process regulated by the transforming growth factor-β (TGF-β) signaling pathway (21), which is also the key driver of continuous decline in renal function. In this case, the affected dog showed no improvement in renal function parameters or clinical symptoms after receiving conventional treatments including fluid therapy and nutritional support. However, after treatment with allogeneic canine AD-MSCs, partial improvement in some biochemical indicators of renal function was observed, along with improvement in clinical signs. This observation may support the potential role of MSC therapy in CKD management and is consistent with the proposed therapeutic mechanisms of MSCs (22). A growing body of evidence suggests that MSCs may exert nephritis-ameliorating and renoprotective effects through multiple potential mechanisms. These proposed mechanisms include: (i) regulation of innate and adaptive immune dysregulation and inhibition of excessive renal inflammatory responses, thereby potentially reestablishing local immune homeostasis (23); (ii) paracrine effects, including secretion of protective cytokines, release of extracellular vesicles, and mitochondrial transfer, which may modulate the microenvironment of injured renal tissue (24); (iii) homing to injured renal tissue via chemokine axes, with potential differentiation into intrinsic renal cells and activation of endogenous repair programs (25); and (iv) inhibition of fibrosis-related signaling pathways, EMT, and abnormal ECM deposition, as well as attenuation of oxidative stress and excessive apoptosis through antioxidant pathways and regulation of apoptosis/autophagy homeostasis (26). In addition, AD-MSCs are generally considered to have advantages such as easy accessibility, strong proliferative capacity, and stable multilineage differentiation potential (27). However, it is important to note that these mechanisms were not directly evaluated in the present case. Therefore, they are presented as potential mechanisms supported by previous literature rather than mechanisms demonstrated in this patient.

The key highlight of this case is the adoption of allogeneic canine AD-MSCs to treat canine CKD, which resulted in an encouraging therapeutic response, consistent with existing research findings on allogeneic MSCs for canine CKD. Studies have suggested that the low immunogenicity of allogeneic MSCs may reduce effectively reduce immune rejection reactions during allogeneic infusion, and the immunomodulatory factors secreted by MSCs may inhibit abnormal systemic immune responses, providing theoretical support for the feasibility of allogeneic stem cell therapy (28). After infusion, the affected dog did not develop immune rejection reactions such as fever and vomiting, nor did it experience other adverse events. However, it is important to note that dexamethasone (0.5 mg/kg IV) was administered before each MSC infusion. As a corticosteroid, dexamethasone may suppress inflammatory responses and affect hematological parameters, and therefore may have confounded the assessment of infusion-related adverse reactions. Consequently, the absence of observed adverse events cannot be solely attributed to the safety of allogeneic AD-MSCs, and the potential confounding effect of dexamethasone should be considered when interpreting these findings. This is consistent with the view that allogeneic MSCs may serve as a potentially safe therapeutic modality for canine CKD, although further studies without concomitant corticosteroid administration are needed to confirm their safety profile. Meanwhile, 3 months after treatment, the blood urea nitrogen (BUN) level of the affected dog returned to the reference range, serum creatinine and phosphorus levels decreased significantly, and body condition score (BCS) was increased. These findings indicate that AD-MSCs can not only alleviate the clinical symptoms of CKD, but also effectively improve renal function and delay disease progression (29).

In combination with existing studies, this case also has several important limitations. First, this is a single-case report with a small sample size and no control group, which precludes verification of the generalizability of this treatment regimen. Second, the concurrent use of conventional treatments (including a renal prescription diet, fluid therapy, and nutritional support) and the administration of dexamethasone before each MSC infusion may have confounded the observed outcomes. Because corticosteroids can suppress inflammation and affect hematological and biochemical parameters, the independent contribution of AD-MSCs cannot be distinguished from these co-interventions. Third, the presence of comorbidities, such as degenerative valve disease, gallbladder mucocele, acute gastroenteritis, and systemic hypertension, may have further confounded the clinical course and response to therapy. Fourth, the lack of renal histopathological examination and biomarker analyses (e.g., cytokines, extracellular vesicles, or fibrosis markers) prevented clarification of the specific molecular mechanisms underlying any observed improvement in renal function. Fifth, due to the retrospective nature of this case, critical IRIS sub-staging parameters, specifically the urine protein-to-creatinine ratio (UPC) and systemic blood pressure, were not sequentially recorded, which limits a fully comprehensive renal evaluation. In the future, it is necessary to increase the sample size, establish a conventional treatment control group, and demonstrate the superiority of AD-MSC therapy over traditional treatment. Long-term follow-up is also needed to evaluate the efficacy and safety of AD-MSC therapy, as canine CKD has a protracted clinical course and may be complicated by progressive renal dysfunction and other comorbidities. Therefore, subsequent studies should extend the follow-up period to assess the long-term impact of AD-MSCs on the quality of life of affected dogs. In addition, future studies should incorporate pathological sectioning, cytokine detection, and other biomarker analyses to further explore the core mechanism by which AD-MSCs delay the progression of canine CKD, so as to provide a theoretical basis for the optimization of the treatment regimen. Collectively, these limitations indicate that the findings of this case report should be interpreted as hypothesis-generating rather than confirmatory, and no definitive causal relationship between AD-MSC therapy and renal function improvement can be established.

In summary, this case suggests that allogeneic AD-MSCs may be a feasible therapeutic approach in the management of severe canine CKD. In this dog, MSC treatment was associated with observed improvements in some renal biochemical parameters and clinical signs, and short-term tolerability was observed; however, dexamethasone was co-administered before each infusion and may have confounded this assessment. The efficacy and safety of allogeneic AD-MSCs cannot be established from a single case, and controlled studies with larger sample sizes and longer follow-up are needed to determine whether AD-MSCs can improve renal function, delay disease progression, and improve the quality of life of dogs with CKD.

## Data Availability

The original contributions presented in the study are included in the article/supplementary material, further inquiries can be directed to the corresponding authors.
